# Comparison of monitoring performance of Bioreactance vs. pulse contour during lung recruitment maneuvers

**DOI:** 10.1186/cc7981

**Published:** 2009-07-28

**Authors:** Pierre Squara, Dominique Rotcajg, Dominique Denjean, Philippe Estagnasie, Alain Brusset

**Affiliations:** 1 ICU, Clinique Ambroise Paré, 27 bd Victor Hugo, 92200 Neuiily-sur-Seine, France

## Abstract

**Introduction:**

This study was designed to test the hypothesis of equivalence in cardiac output (CO) and stroke volume (SV) monitoring capabilities of two devices: non invasive transthoracic bioreactance (NICOM), and a pulse contour analysis (PICCO PC) coupled to transpulmonary thermodilution (PICCO TD).

**Methods:**

We included consecutive patients of a single ICU following cardiac surgery. Continuous minute-by-minute hemodynamic variables obtained from NICOM and PICCO PC were recorded and compared in 20 patients at baseline, during a lung recruitment maneuver (20 cmH_2_O of PEEP) and following withdrawal of PEEP. PICCO TD measurements were also determined. We evaluated the accuracy of these two technologies at baseline using PICCO TD as reference and we estimated the precision by the fluctuation around the mean value (2SD/mean). Then, we assessed time response, amplitude response and reliability for detecting expected decreases when PEEP was applied. Type I and type II errors were analyzed.

**Results:**

CO values (PICCO TD) ranged from 1.6 to 8.0 L.min^-1^. At baseline, CO values were comparable for NICOM, PICCO PC and PICCO TD: 5.0 ± 1.2, 4.7 ± 1.4 and 4.6 ± 1.3 L.min.^-1^, respectively (NS). Limits of agreements with PICCO TD were 1.52 L.min.^-1 ^for NICOM and 1.77 L.min.^-1 ^for PICCO PC, NS. The 95% statistical power gives an equivalence with a threshold of 0.52 L.min.^-1 ^for NICOM vs. PICCO PC. The CO precision was 6 ± 3% and 6 ± 5% for NICOM and PICCO PC, respectively, NS. When PEEP was applied, CO was reduced by 33 ± 12%, 31 ± 14% and 32 ± 13%, for NICOM, PICCO PC and PICCO TD, respectively (NS). Time response was 3.2 ± 0.7 minute for NICOM vs. 2 ± 0.5 minute for PICCO PC (NS). SV results were comparable to those for CO.

**Conclusions:**

Although limited to 20 patients, this study has enough power to show comparable CO and SV monitoring capabilities of Bioreactance and pulse contour analysis calibrated by transpulmonary thermodilution.

## Introduction

Cardiac output (CO) and stroke volume (SV) are fundamental physiologic variables used for diagnosis and guiding therapy in many clinical settings. The most widely trusted technology for measuring these variables is still bolus thermodilution [1,2]. However, this technology only allows for measurements at discrete moments in time and measurements are usually obtained only a few times per day. In addition, bolus thermodilution is invasive, costly and significantly time consuming for highly trained medical personnel when placing a pulmonary artery catheter and for its subsequent care. Increasingly, it has been recognized that monitoring and treatment protocols should be based on frequent customization of fluid and drug treatment, relying on continuous monitoring and measurement of dynamic hemodynamic responses vs. snapshots of CO or filling pressures [3-5]. This has led to the emergence of several continuous, less invasive and easier to use modalities.

Clearly, the information and clinical utility of technologies that provide discrete measurements of CO obtained from bolus thermodilution and those that offer continuous CO monitoring capabilities are different [6]. The criterion by which a CO measurement device is evaluated is primarily the accuracy of measurements quantified by the averaged bias and the inter-patient variability of bias versus a reference method [7]. However, for continuous monitoring devices, time-dependent criteria such as precision (variability over time due to random error of measurements), time response, amplitude response, and ability to detect clinically meaningful changes are of primary importance [8].

A new, continuous, noninvasive CO monitoring (NICOM^®^) device based on analysis of transthoracic Bioreactance^® ^has been introduced recently [9]. Bioreactance is the analysis of the variation in the frequency spectra of a delivered oscillating current that occurs when the current traverses the thoracic cavity, as opposed to the traditional bioimpedance, which relies only on analysis of changes in signal amplitude. Three prior validation studies comparing bioreactance to bolus thermodilution (PAC) [10], continuous thermodilution (PAC CCO) [8], and arterial pulse wave analysis (VIGILEO) [11] showed that bioreactance was comparable with these other methods based on the criteria noted above. The aim of the present study was to compare the CO monitoring capabilities of the bioreactance technology with those of a pulse contour (PC) based technology coupled with transpulmonary thermodilution (TD) during a hemodynamic challenge, namely lung recruitment maneuvers, in post-cardiac surgery patients.

## Materials and methods

The protocol was approved by our institutional review board. Informed consent was obtained from each patient. A PICCO+^® ^system (Pulsion Medical System, Munich, Germany) was inserted to determine CO from both TD and PC in patients in whom hemodynamic monitoring was indicated according to our standard clinical practice. The arterial line was inserted in the radial artery [12]. Noninvasive bioreactance CO monitoring was obtained using the NICOM^® ^system (Cheetah Medical Inc., Portland, OR, USA) described previously [13]. All PICCO+ and NICOM continuous variables were recorded simultaneously using a computer data logger that recorded data at one minute intervals.

Data were obtained from 20 post-cardiac surgery, intubated and mechanically ventilated patients at a single center. Patients were studied during a period of hemodynamic stability just prior to weaning off mechanical ventilation [14] and there were no changes in therapy during the protocol. All patients had post-operative echocardiography to check the absence of tricuspid, mitral or aortic valve insufficiency. The PICCO+ and NICOM devices were calibrated as recommended by the manufacturers. For PICCO+ this consisted of obtaining three concordant (<20% differences) bolus TDs, automatically used by the system to calibrate the PC analysis. The NICOM device was calibrated during a five-minute auto-calibration cycle. In each patient, a lung recruitment maneuver, consisting of applying 20 cmH_2_O positive end expiratory pressure (PEEP) for 10 minutes, was introduced. PEEP was reduced to 15 cmH_2_O when poorly tolerated. For both PICCO PC and NICOM, CO, SV and heart rate (HR) trends were recorded minute by minute for 10 minutes prior to PEEP (baseline), during a 10-minute period of PEEP application, and for 10 minutes following withdrawal of the PEEP, so that the total number of measurements for inter device comparison was 30. The reference CO values were obtained by three concordant bolus PICCO TDs after the initial calibration just before PEEP during the baseline period, during the application of PEEP, and after withdrawal of PEEP. This automatically led to recalibrate the PICCO PC signal three times during the 30-minute period of the protocol.

Accuracy (i.e., bias) was quantified by the difference between values assessed by the two monitoring devices and PICCO TD. We compared CO, SV and HR values at the following three time periods: the average values during the three minutes immediately before PEEP (baseline, protocol minutes 8, 9 and 10); during the three-minute period of lowest CO values during PEEP (between protocol minutes 10 and 20); and during the final three minutes following withdrawal of PEEP (protocol minute 28, 29 and 30).

Precision (the variability due to random error of measurements) is most easily quantified during periods of stable CO, such as the 10 minute baseline period of the protocol. During this period, we obtain 10 CO measurements. From these 10 measurements, we determined the mean and standard deviation (SD) of the measurement. Precision is then determined as two × SD/mean.

Time response was quantified as the delay between the PEEP application and the point when the minimum CO was obtained. Amplitude response was assessed as the difference between the average baseline CO and the minimum CO recorded during PEEP.

To test globally the ability to detect significant CO changes and the equivalence of monitoring capabilities of NICOM and PICCO PC we calculated the cross correlations between the two technologies, including the 30 recorded minutes of the protocol.

### Data analysis

Studied values were different according to the studied criteria (see above). For accuracy of continuous monitoring technologies (NICOM and PICCO PC), average baseline values from three minutes were compared with the reference (PICCO TD) using linear regressions analysis with coefficient of determination (R) derivation and comparison with identity line. Bias was calculated as the difference in mean values (reported as inter patient mean ± SD). Relative error (absolute value of bias/mean) and limits of agreements (± 2 × SD about the differences of mean values between two modalities) were determined [15]. The proportion of patients for which the bias was acceptable according to the criteria determined by Critchley and Critchley [16] was also reported. Student t-tests were used to reject the null hypothesis. However, absence of evidence is not evidence of absence [17,18]. A non-significant difference between two devices does not take into consideration type II errors. To test equivalence of bias, we compared the confidence interval of the differences and estimated the threshold of difference in means that gives a statistical power of 95% (for t-test).

## Results

**Figure 1:**
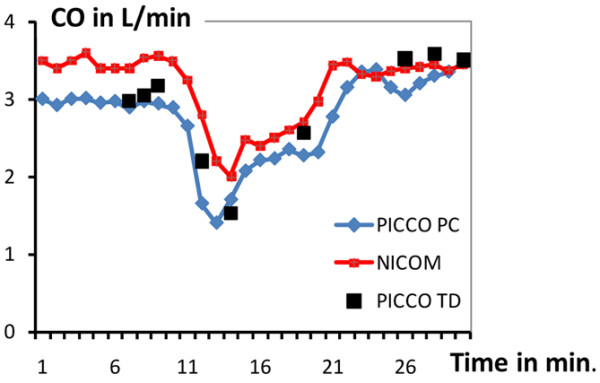
Typical original recordings of PICCO PC, PICCO TD and NICOM during the 30-minute study protocol. Recordings included the baseline period (minutes 0 to 10), during positive end expiratory pressure (PEEP; minutes 10 to 20), and following PEEP removal (minutes 20 to 30). CO = cardiac output.

The study participants included 14 men and 6 women, age 69 ± 13 years. Nine patients received coronary grafts, seven patients underwent valve replacements (including two mitral valve repairs) and four patients had mixed interventions. Left ventricular ejection fraction was 52 ± 9%. At the time of the protocol, none of the patients had circulatory failure or acute pulmonary edema (partial pressure of arterial oxygen/fraction of inspired oxygen = 281 ± 58 mmHg). Seven patients were receiving a moderate degree of inotropic support (five patients were receiving dobutamine 5 μg/kg/min, two patients were receiving adrenaline 0.25 mg/h, no patient received noradrenaline). Figure 1 shows a typical example of the minute-by-minute CO values from NICOM and PICCO PC during the 30-minute protocol. As seen, CO values are comparable at baseline, decrease rapidly with the introduction of PEEP, reaching similar minimum values within three to four minutes, and rebound to a plateau after another two to three minutes. Following withdrawal of PEEP, at protocol minute 20, CO returns to near the original baseline value. During the protocol (baseline, PEEP and return to baseline), the HR was quite stable: 93 ± 22, 95 ± 24, and 92 ± 19 beats/min, respectively (not significant (NS)); the corresponding mean systemic pressure was 78 ± 13, 63 ± 18, and 79 ± 14 mmHg, respectively (*P *< 0.01 for pressure during PEEP application vs. baseline and return to baseline).

For the data as a whole, CO values were comparable for NICOM, PICCO PC, and PICCO TD at baseline (5.03 ± 1.16, 4.73 ± 1.44, and 4.61 ± 1.26 L/min, respectively; NS), at their nadir following introduction of PEEP (3.34 ± 0.83, 3.23 ± 0.91, and 3.22 ± 0.89 L/min, respectively), and following recovery from PEEP (4.98 ± 1.11, 4.88 ± 1.50, and 4.87 ± 1.03 L/min, respectively). When the 60 CO values (pre-PEEP, PEEP, and post-PEEP) were included, confidence interval of the difference was -1.5 to 1.9 for NICOM – PICCO TD, -1.9 to 1.9 for PICCO PC – PICCO TD, and -2.2 to 2.5 for NICOM – PICCO PC. The 95% statistical power gives a mean CO difference threshold of 0.77, 0.29, and 0.52 L/min for NICOM vs. PICCO TD, PICCO PC vs. PICCO TD and NICOM vs. PICCO PC, respectively, corresponding approximately to 0.44, 0.16, and 0.25 L/min/m^2^. Table 1 summarizes additional statistics comparing PICCO-PC and NICOM with PICCO-TD during the two stable hemodynamic periods (i.e., the baseline period and the post-PEEP period). The different criteria of CO accuracy were comparable.

**Table 1:** Inter technology agreements based on 20 values for each technology

	NICOM vs. PICCO TD	PICCO PC vs. PICCO TD	NICOM vs. PICCO PC
**Bias**	0,26	0,04	0,22
**R**	0,76	0,77	0,55
**RE**	0,12	0,11	0,18
**LOA**	1,52	1,77	2,41
**CV**	0,31	0,38	0,49
**C&C**	0,93	0,93	0,80

Values of cardiac output obtained during positive end expiratory pressure were excluded from this analysis to minimize the impact of differences in time responsiveness between the devices.

R = coefficient of determination for inter-technologies comparisons; RE = averaged value of relative error (absolute bias/mean); LOA = limits of agreements of the Bland and Altman representation (two × standard deviations of the difference) in L/min; CV = coefficient of variation (two × standard deviation/mean); C&C = percentage of the differences inside the 30% limit of acceptability as suggested by Critchley and Critchley [16].

**Figure 2:**
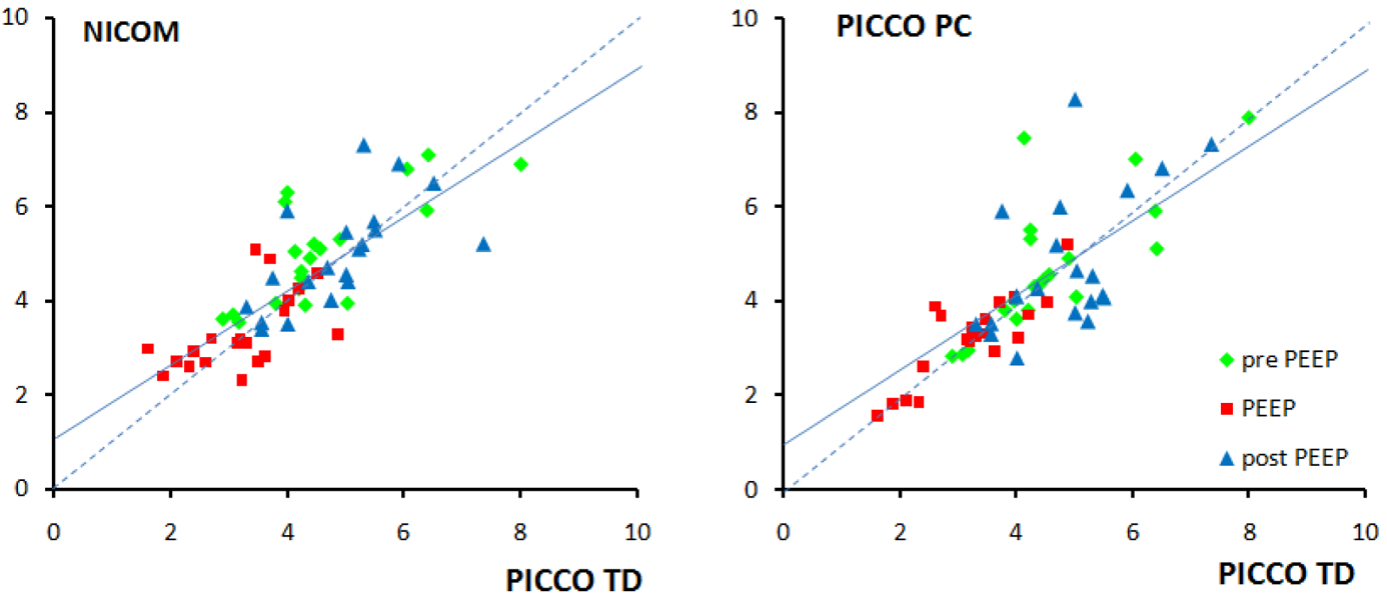
Comparison of cardiac output measured by NICOM and PICCO PC in comparison of PICCO TD. The three different colours represent baseline, positive end expiratory pressure (PEEP) application, and return to baseline. The regression lines did not differ significantly from the line of identity (PICCO TD vs. NICOM: y = 1.2 + 0.77x, r = 0.77; PICCO TD vs. PICCO PC: y = -0.5 + 0.9x, r = 0.79).

**Figure 3:**
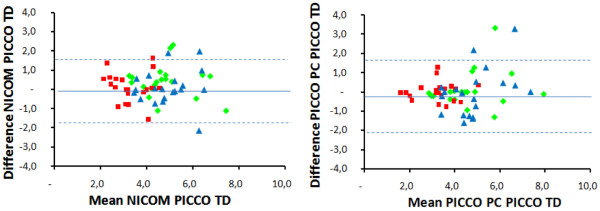
Bland-Altman plots comparing NICOM and PICCO PC to PICCO TD. The three different colours represent baseline, positive end expiratory pressure application, and return to baseline. Mean bias and limits of agreements are given in Table 1. Mean bias and limits of agreements are 0.22 ± 1.67 L/min/m for NICOM and 0.01 ± 1.86 L/min/m for PICCO PC.

In all patients, the three technologies detected a decrease in CO when PEEP was applied. CO was reduced by 33 ± 13%, 31 ± 15%, and 35 ± 13%, for NICOM, PICCO PC, and PICCO TD, respectively (NS). The changes in CO noted between different periods of the protocol were mainly due to changes in SV with minimal changes in HR (see below). A detailed accounting of the comparisons are shown in Figure 2 (comparing NICOM and PICCO PC with PICCO TD, with further statistical details provided in the figure legend) and in the respective Bland-Altman graphs in Figure 3. As shown, the bias was negligible and the limits of agreement to PICCO TD were comparable for NICOM and PICCO PC, amounting to about ± 2 L/min.

Precision of the NICOM and PICCO-PC CO were comparable: 5.6 ± 2.9% and 6.1 ± 4.5% for NICOM and PICCO PC, respectively. Time to minimum CO value following introduction of PEEP (our measure of time responsiveness) was 2.6 ± 0.5 minute for PICCO PC and 3.2 ± 0.7 minute for NICOM (NS) and a reduction of CO was detected by both methods within one minute.

The cross correlations between PICCO PC and NICOM was r = 0.62 ± 0.15 (extremes 0.22 to 0.86). In six patients it was <0.5. In four of these six patients, the PEEP-induced CO change was small (<20%).

SV were very close at baseline for NICOM, PICCO PC, and PICCO TD: 53 ± 21, 50 ± 26, 49 ± 27 mL/beat, respectively (NS). Precision of the devices to measure SV was identical to that noted above for CO: 8 ± 7% and 9 ± 5% for NICOM and PICCO PC SV, respectively. During PEEP, SV was reduced similarly to CO in all patients and all technologies: 31 ± 13%, 31 ± 19%, 32 ± 17% for NICOM, PICCO PC, and PICCO TD, respectively (NS). There was complete inter-technology agreement in HR detection: 94 ± 29 vs. 95 ± 29 beats/min at baseline (NS). HR increased in eight patients, was unchanged in three patients, and decreased in nine patients. The average change in HR was a small decline: 0.4 ± 11 vs. 1.1 ± 12 beats/min for NICOM and PICCO, respectively (NS).

## Discussion

CO and SV are fundamental variables for assessing circulatory disorders and their response to therapeutic interventions. The ability to continuously monitor these variables improves our capabilities of tracking diseases and optimizing therapy. Using a standardized intervention (i.e., PEEP challenge) known to result in a rapid reversible reduction of CO [19,20], we have demonstrated that PICCO PC and NICOM have equivalent CO monitoring capabilities, including the ability to detect directional changes in CO.

We used PICCO TD as the reference for comparisons of absolute CO values and changes in CO detected by NICOM and PICCO PC. Although transpulmonary TD provided by PICCO TD has received significant interest [21], it is not widely accepted as a reference technology for CO measurement [22,23]. Nevertheless, this technique allowing periodic recalibrations to increase the accuracy of the continuous monitoring is widely used and the results of the present study therefore assessed performance of the NICOM relative to PICCO TD, not necessary to a universally accepted gold standard.

When compared with PICCO TD, the averaged bias and the coefficient of variation of NICOM and PICCO PC were comparable. According to the criteria of Critchley and Critchley [16], the bias was acceptable in 93% of the cases. Despite a relatively small number of patients, this study was adequate to show a practical equivalence in accuracy between the three technologies. The threshold for a statistical power of 95% is always less than 0.5 L/min/m^2^. The threshold between PICCO PC and PICCO TD was necessarily the smallest because PICCO PC was automatically recalibrated directly against PICCO TD just prior to starting the protocol, then at baseline, during PEEP, and after withdrawal of PEEP. In comparison, the NICOM device is completely noninvasive, self-calibrating and utilizes calibration factors determined in prior studies as detailed previously [13].

As noted above, devices intended for continuous hemodynamic monitoring are required not only to provide acceptable estimates of CO and SV, but are also expected to manifest appropriate precision and responsiveness during times of changing hemodynamic performance. Good precision and responsiveness are thus essential in order to quickly detect any directional change and provide reliable and rapid indications of clinically meaningful changes that would require medical intervention. Within the limits of the present study, it can be concluded that NICOM and PICCO PC fulfill this criterion. When discrete measurements are compared with a gold standard giving the true value, precision, namely the variability of measurements due to random error of measurement can be assessed by the variability of bias. However, when comparing monitoring tools when none of them can be considered as a gold standard, precision is better estimated as the variability around a stable trend line slope [6,8]. That is why we chose to compare PICCO PC and NICOM precision during the baseline period of our study.

Although restricted to a unique hemodynamic test, our study employed a challenging intervention for any CO monitoring system because it imposes sudden, relatively complex changes in right and left ventricular afterloads and preloads due to impact on venous return and extracardiac pressures. When PEEP is applied, it results in a sudden decrease in CO then a sudden increase when PEEP is removed. Despite these opposite and challenging conditions, our results are consistent with previous studies [8,11], so it is reasonable to generalize the findings to other hemodynamic challenges. PICCO PC technology is based on a peripheral PC analysis technology, therefore in a theoretical aortic flow-impedance model and in a transfer function deriving this proximal aortic flow-impedance relation from a peripheral arterial wave signal. NICOM technology is based on the relation between changes in chest bioreactance and changes in aortic volume from which SV is extrapolated [13]. A sudden increase in intra-thoracic pressure such as high PEEP application decreases the aortic compliance and may change both the transfer function of the PICCO PC and the SV/aortic volume relation of the NICOM. In addition, very high pulmonary pressure may lead the NICOM to overestimated the CO [8]. In this limited number of patients, it does not appear that these changes may have strong consequences on the CO accuracy.

## Conclusions

Although occasional discordances may occur in CO values assessed by transthoracic bioreactance and PC arterial wave analysis, precision, time, and amplitude responsive, and the ability to detect significant CO changes were equivalent and acceptable for both technologies. Because NICOM is totally noninvasive, it can markedly expand the number of patients in which accurate continuous CO monitoring is possible.

## Key messages

• Chest bioreactance is equivalent to PICCO PC in terms of precision, time, and amplitude response.

• The threshold for bias equivalence in this study was 0.52 L/min.

## Abbreviations

CO: cardiac output; HR: heart rate; NS: not significant; PC: pulse contour; PEEP: positive end expiratory pressure; SD: standard deviation; SV: stroke volume; TD: thermodilution.

## Competing interests

PS is a consultant for Cheetah med. All other authors declare that they have no competing interests.

## Authors' contributions

PS, AB, and PE designed the study. DR and DD collected the data. PS made the statistical analysis and wrote the draft of the manuscript. All authors finalized and approved the manuscript.
